# 现代仪器分析课程开放性实验：生物质衍生功能材料的制备及其对水环境中抗生素的富集应用

**DOI:** 10.3724/SP.J.1123.2025.10028

**Published:** 2026-09-08

**Authors:** Xiaoyun LEI, Lingxia JIN, Xiaoping WU

**Affiliations:** 1. 陕西理工大学化学与环境科学学院，陕西 汉中 723001; 1. School of Chemistry and Environmental Science，Shaanxi University of Technology，Hanzhong 723001，China; 2. 福州大学化学学院，福建 福州 350116; 2. College of Chemistry，Fuzhou University，Fuzhou 350116，China

**Keywords:** 现代仪器分析, 开放性实验, 生物质衍生聚合物, 固相萃取, 水体污染物, modern instrumental analysis, open-ended experiment, biomass-derived polymer, solid-phase extraction, water pollutants

## Abstract

为培养化学及相关专业高年级本科学生的系统思维与创新能力，本项目设计了一个以生物质衍生功能材料的制备、表征与应用为主线的开放性实验，结合了生物炭材料制备、聚合物合成等关键实验操作，并采用扫描电子显微镜、红外光谱及水接触角测试对材料结构与亲疏水性进行表征；在应用环节中，进一步利用自制材料吸附实际水样中的糖肽类抗生素，并结合高效液相色谱法完成定量分析。在实验过程中，学生将深化对多学科知识的融合运用，学习和掌握从样品前处理到仪器分析的全分析流程技能，提升材料制备、仪器操作与数据分析的综合能力，通过该实践切实理解分析技术在环境治理和监测中的应用，全面提高发现和解决复杂化学问题的创新素质。此外，这一开放性实验的开展，也有助于推进生物质材料资源化利用，符合当前低碳经济的需求。

## Abstract (en)

To cultivate systematic thinking and innovation capabilities in senior undergraduate students majoring in chemistry and related fields， this project proposes an open-ended experiment that focuses on the preparation， characterization， and application of biomass-derived functional materials. The experiment incorporates essential procedures such as the preparation of biochar materials and the polymer synthesis. Characterization of the material’s structure and hydrophilicity/hydrophobicity is performed using scanning electron microscopy， infrared spectroscopy， and water contact angle measurements. During the application phase， the self-prepared materials are utilized to adsorb glycopeptide antibiotics from real water samples， followed by quantitative analysis using high performance liquid chromatography. Throughout the experiment， students will enhance their ability to integrate multidisciplinary knowledge， master skills across the entire analytical workflow—from sample pretreatment to instrumental analysis—while also improving their competencies in material preparation， instrument operation， and data analysis. Through this hands-on practice， students will develop a practical understanding of the application of analytical technologies in environmental remediation and monitoring， thereby strengthening their innovative capacity to identify and solve complex chemical problems. Furthermore， the implementation of this open-ended experiment contributes to advancing the resource utilization of biomass materials， aligning with the demands of a low-carbon economy.

现代仪器分析是一种以物质的物理和化学性质为基础建立起来的分析方法，主要依托基于光谱、电化学、色谱或质谱等技术的先进科学仪器，系统开展物质的定性、定量、形态分析及结构解析，已成为化学、材料、物理、医药、生物等多学科交叉领域的关键研究手段，对推动科学进步与技术突破具有不可替代的作用^［1］^。为培养学生掌握仪器原理、操作技能与数据分析能力，提升其科研素养和解决实际问题的能力，国内外高校普遍将现代仪器分析实验课程纳入化学及相关专业的本科生教学体系^［2，3］^。然而，传统的实验教学以验证性内容为主，学生遵循既定步骤操作仪器，缺乏应对复杂科研问题所需的系统性训练。传统教学模式主要依赖教师示范和学生分批上机操作，难以充分激发学生的创新思维与科研自主性，限制了教学效果的进一步提升。因此，构建一门开放性实验课程，将功能材料的合成、表征和应用等内容融入现代仪器分析实验教学，促进本科生在实验中探讨学习和互相协作，具有重要的现实意义。此类课程设计不仅能够增强学生的综合实践能力与团队协作意识，也为他们未来职业发展或学术深造打下坚实基础。

水体污染物是指导致水体水质、生物群落及底泥恶化的各类有害物质，尤其包括有机毒物、耗氧有机物和无机化学物。这些物质常伴随人类活动排放到自然环境中，不仅引发严重的环境污染，还可能对人体健康构成威胁^［4］^。其中，抗生素作为典型的痕量污染物，易在环境水体中残留，对公共健康造成潜在威胁。因此，建立水体中痕量抗生素的高灵敏分析方法，实现污染物的及时监测与识别，对保障环境安全具有重要意义^［5］^。然而，环境水样基质复杂，常含有悬浮颗粒、溶解性有机物及无机盐等组分，且污染物浓度通常处于ng/L~μg/L的较低水平，难以直接进行分析检测，样品前处理成为了不可或缺的环节^［6］^。目前，固相萃取（SPE）技术因其操作简便高效、灵敏度高、易于实现自动化及现场应用等优势，已成为环境水样中化学污染物检测和筛查的主要前处理方法^［7-9］^。SPE是利用吸附剂对目标物的选择性吸附作用实现分离和富集，目前常用吸附剂有硅胶基质、无机基质、有机聚合物等。然而，在保证吸附剂萃取效率的前提下，如何减少前处理和化学分析过程中的目标物损耗以及对环境造成的二次影响，仍是SPE技术面临的重要挑战。

近年来，SPE技术的一个重要发展方向是引入绿色吸附剂，如可生物降解材料、农业废弃物或无毒组分，以逐步替代传统吸附材料^［10，11］^。这类材料通常具有高孔隙率、低流动阻力以及易于进行表面化学或物理修饰等优点，极大拓展了其在SPE中的应用潜力。其中，以生物质为前驱体制备的生物炭（BC）作为一种典型的碳基材料受到广泛关注，其优势在于来源可持续（常利用废弃物制备）、比表面积大、表面含氧官能团丰富，这些特性对实现高效吸附十分重要^［12，13］^。然而，纯BC因其粒径较小，直接用作SPE吸附剂时易造成柱体堵塞，且在装填和上样过程中容易发生流失，影响萃取结果的稳定性与重现性；此外，纯BC材料通常循环使用性能较差^［14］^。因此，采用BC作为基底，通过表面负载纳米材料来构建聚合物骨架复合吸附剂，能够在增强复合材料机械强度和结构稳定性的同时进一步提升其吸附性能，是当前绿色SPE吸附剂开发中一个值得深入探索的研究方向。

基于上述研究背景与教学需求，笔者所在团队设计了一项开放性实验，将生物质衍生功能材料的制备、表征及其在水污染物SPE技术中的应用纳入现代仪器分析实验教学。在教师指导下，学生通过查阅文献自主设计实验方案，并逐步完成复合材料的合成、结构表征、吸附性能评估以及实际水样中糖肽类抗生素污染物的色谱分析全过程。本实验选择万古霉素、替考拉宁作为目标分析物，二者均含有七肽核心骨架、糖基化修饰单元及多个芳香环（见图1）。基于以上结构特点，本研究制备的生物质衍生复合材料可通过多种作用力实现对目标物的高效吸附。实验内容涵盖材料合成、光谱和显微分析、接触角测量和色谱检测等多种技术手段，融合了绿色吸附材料设计与样品前处理等前沿研究方向。该实验不仅使学生掌握多种仪器操作方法，更引导其关注环境分析和绿色化学领域的先进材料与可持续发展理念，有助于培养学生的科研创新意识、综合实践能力与社会责任感，为将来从事环境化学、分析化学等相关领域的研究或工作奠定扎实基础。

**图1:**
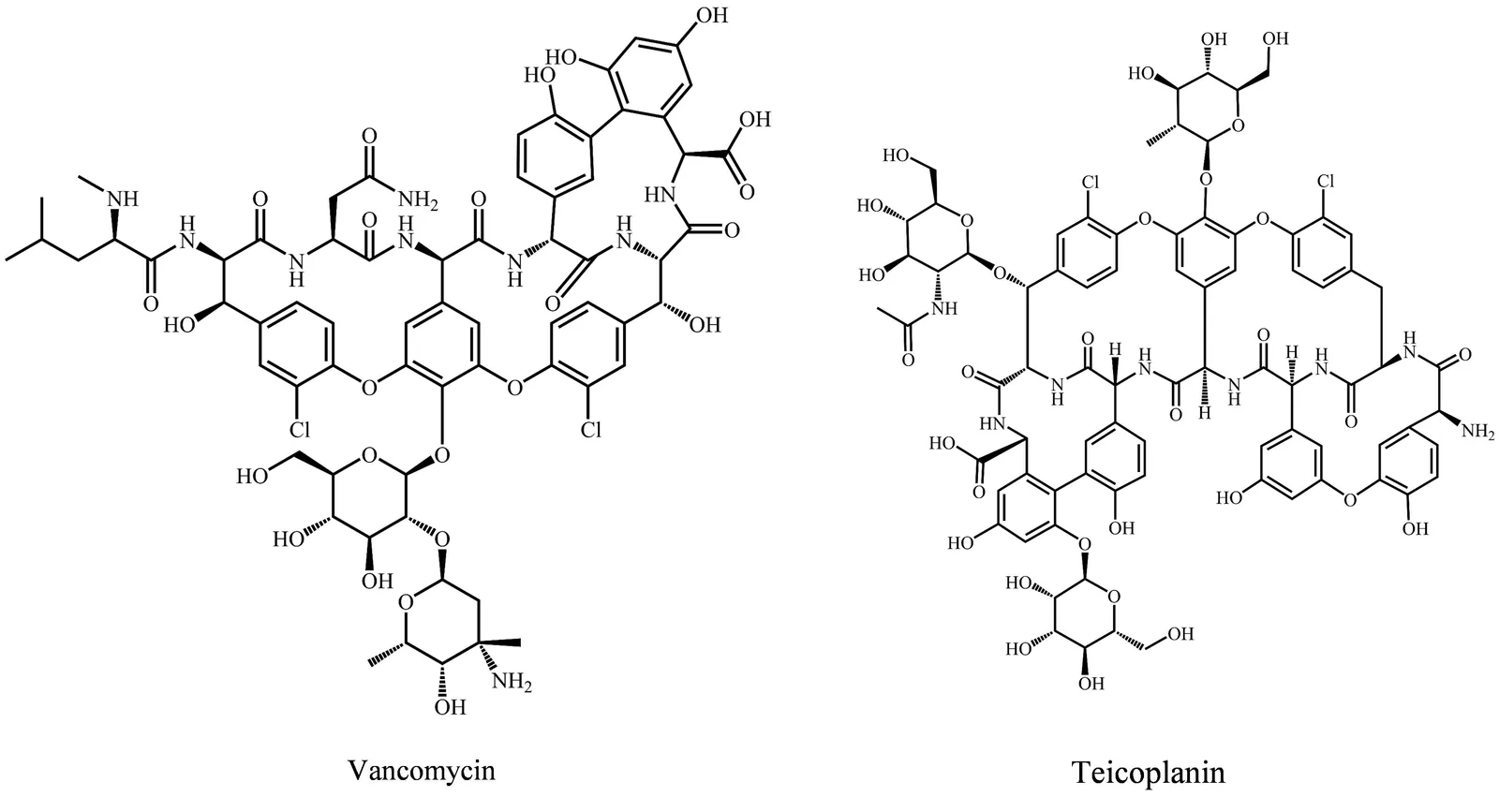
万古霉素和替考拉宁的化学结构

## 1 实验部分

### 1.1 仪器与试剂

高效液相色谱系统（SPD-M20A，岛津，日本），主要包括：LC-20AD双泵、SIL-20A自动进样器、SPD-M20A二极管阵列检测器、CTO-20AC柱温箱；傅里叶变换红外光谱仪（FTIR，Nicolet iS50型）和场发射扫描电子显微镜（SEM，Verios G4型）（Thermo Fisher Scientific，美国）；接触角测量仪（DSA25S型，KRÜSS GmbH，德国）；高压反应釜（GSHA型，威海化工机械有限公司，中国）；马弗炉（KSL-1700X型，合肥科晶材料技术有限公司，中国）；高速离心机（TGL-20M型，湖南湘仪实验室仪器开发有限公司，中国）；真空干燥箱（DZ-1BC1V型，天津市泰斯特仪器有限公司，中国）。

抗生素万古霉素与替考拉宁标准品（纯度≥98%）购自Sigma-Aldrich公司。POSS-环氧树脂（POSS-epoxy，分析纯）购自长沙巴溪仪器有限公司。甲醇、甲酸（FA）、乙酸（HAc）、三氟乙酸（TFA）、磷酸（PA）、乙腈、聚乙烯亚胺（PEI，*M*_w_=600）、聚乙二醇200（PEG 200）、聚乙二醇10000（PEG 10000）、2，2′-偶氮二异丁腈（AIBN）均为分析纯，购自上海麦克林生化科技有限公司。实验所用生物质原料，如咖啡渣、秸秆、花生壳等，取自当地农产品加工废弃物。

### 1.2 标准溶液的配制

准确称取5.0 mg抗生素标准品，加入5.0 mL纯水，超声处理15 min，配制成1.0 mg/mL标准储备液，于‒20 ℃避光保存。移取适量标准储备液，甲醇-水（9∶1，体积比）溶液进行稀释，配制标准工作溶液。

### 1.3 生物质衍生聚合物材料的制备

如图2所示，任选一种生物质原料（如秸秆）放入马弗炉中于300 ℃ 煅烧30 min，冷却后经去离子水清洗，随后放入80 ℃烘箱中干燥24 h，粉碎并过100目筛备用，得到BC。准确称取POSS-epoxy 50 mg、PEI 20 mg、PEG 10000 20 mg、PEG 200 90 mg、BC 20 mg溶于180 mL乙腈中，超声处理15 min使其分散均匀。将上述混合液置于水浴锅中，于60 ℃反应16 h。反应结束后，将产物研磨，经真空干燥箱中60 ℃过夜干燥后，得到BC@PEI-POSS聚合物材料。

**图2:**
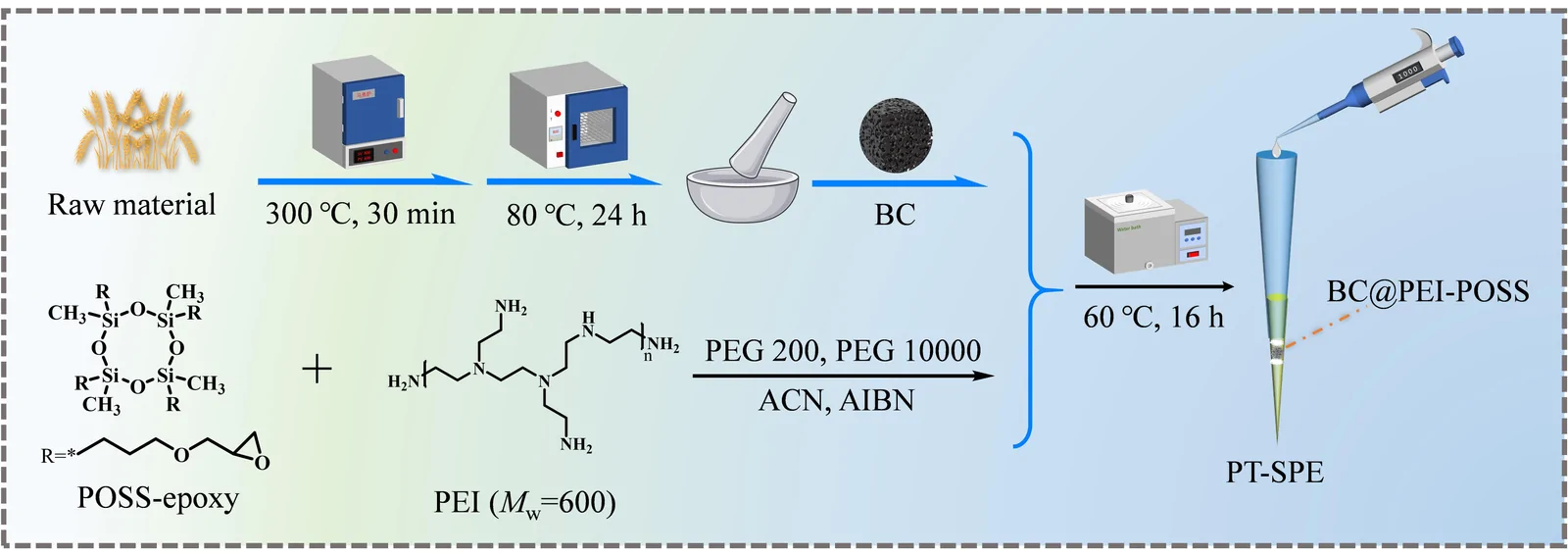
生物质衍生功能材料BC@PEI-POSS的制备过程示意图（以秸秆为例）

### 1.4 移液器吸头固相萃取（PT-SPE）方法

#### 1.4.1 PT-SPE装置

采用移液器吸头构建PT-SPE装置：将10 mg BC@PEI-POSS聚合物材料填充至200 μL移液器吸头中，两端使用脱脂棉进行固定，再将1 000 μL吸头尖端切除适当长度后与之连接。每次使用前均使用1 mL甲醇进行活化。

#### 1.4.2 实际水样PT-SPE过程

实际水样经0.22 μm水系微孔滤膜（混合纤维素酯）过滤，以去除悬浮颗粒物。随后，将1.0 mL过滤后的水样与3.0 mL甲醇-水溶液（9∶1，体积比）混合，并超声处理15 min。取3.0 mL所得样品溶液加入PT-SPE装置，以4 000 r/min离心10 min，使目标抗生素被吸附剂保留，弃去上样流出液。随后，向吸附剂中加入300 μL甲酸-水溶液（0.5∶99.5，体积比），以6 000 r/min离心10 min，收集洗脱液用于后续分析。所有水样在上样前均用甲醇-水溶液（9∶1，体积比）按上述比例混合，以保证两种抗生素的质量浓度分别处于各自的线性范围内，随后采用相同方法进行PT-SPE处理。

### 1.5 HPLC-UV分析

色谱柱为Caprisil C18-X（100 mm×2.1 mm，1.8 μm，Waters，美国），流动相A相为0.1%甲酸水溶液，B相为0.1%甲酸甲醇，柱温保持在28 ℃，单次进样量为5 μL。梯度洗脱程序具体设置如下：0～0.5 min，5%B；0.5～1.5 min，5%B～15%B；1.5～3.0 min，15%B～90%B；3.0～3.5 min，90%B；3.5～3.6 min，90%B～5%B；3.6～15.0 min，5%B。流速为1.0 mL/min。

## 2 结果与讨论

### 2.1 生物质衍生功能材料的表征

#### 2.1.1 显微结构表征

采用SEM对生物炭和所制备的BC@PEI-POSS聚合物材料进行微观结构表征。如图3a和3b所示，生物炭表面纹理粗糙，伴有明显的片状特征，具有丰富的孔隙结构，但其孔径结构均匀性较差。相比之下，BC@PEI-POSS聚合物材料（图3c和3d）展现出良好的三维网络结构，其中生物炭本身所具有的多孔结构为聚合物材料内部均匀形貌的形成提供了良好基础，PEI分子中的伯氨基对POSS-epoxy单体上的环氧基团发起亲核进攻，引发环氧开环，形成球状交联结构。该结构内部具有相互贯通的大孔和介孔，不仅为流体扩散提供了高效的传质通道和良好的渗透性能，同时提供了大量的可吸附位点，从而有利于在萃取过程中实现对目标物的快速传输与高效吸附。

**图3:**
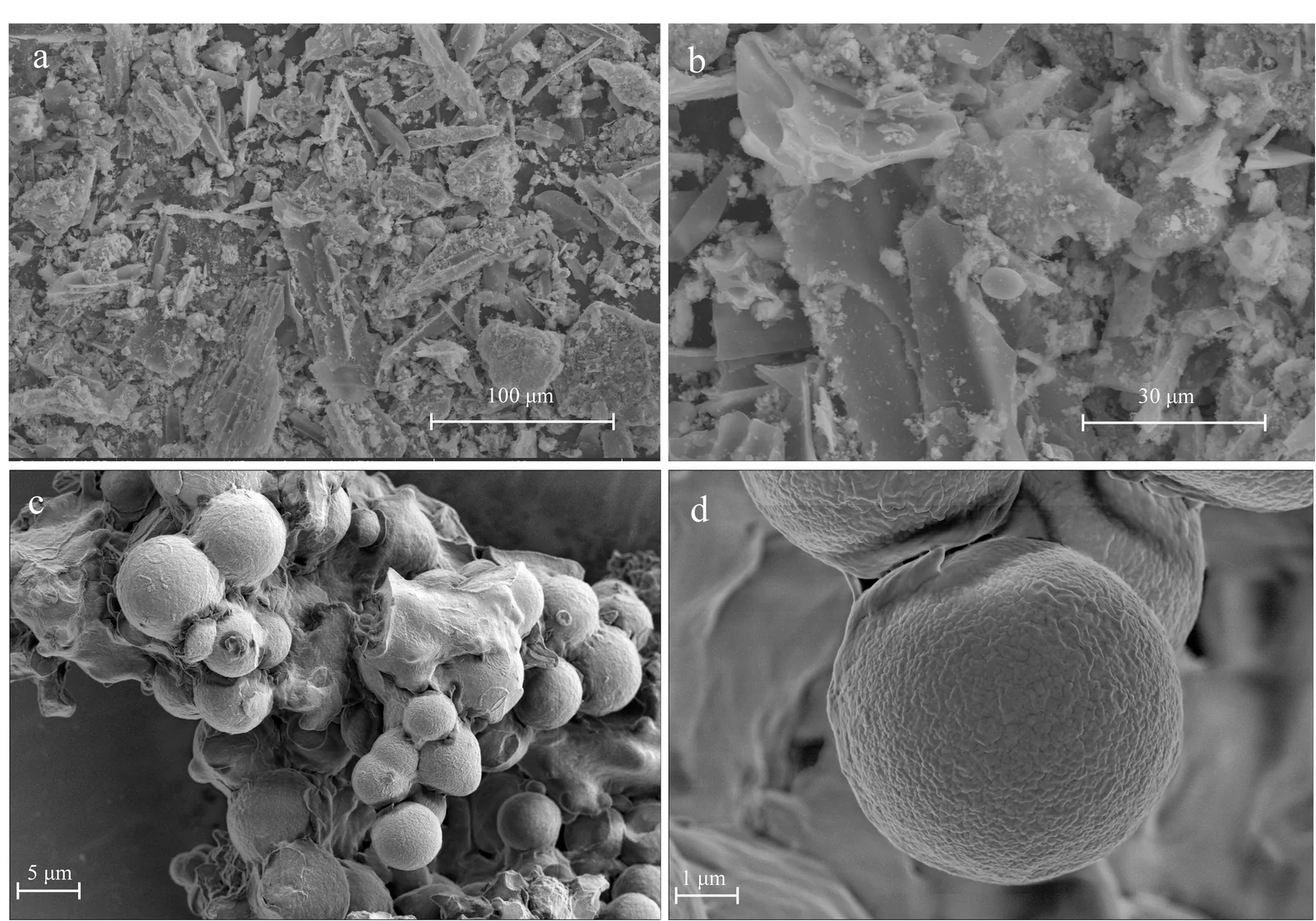
生物炭和生物质衍生功能材料BC@PEI-POSS的SEM图

#### 2.1.2 红外光谱分析

采用FTIR对聚合物材料的化学结构进行表征。如图4a所示，在948 cm^‒1^附近出现的吸收峰归属于环氧基团（C-O伸缩振动），在2 905 cm^‒1^处的吸收峰可能归属于POSS-epoxy单体中烷基链的C-H伸缩振动，1 107 cm^‒1^附近出现的强吸收峰为Si-O-Si不对称伸缩振动。从图4b中可知，948 cm^‒1^处的环氧基特征峰明显减弱甚至消失，同时在3 769 cm^‒1^附近出现O-H伸缩振动峰，表明POSS-epoxy中的环氧基团与PEI的伯氨基发生开环反应，生成羟基及C-N键。进一步地，在最终合成的BC@PEI-POSS材料（曲线c）中，可以观察到3 744 cm^‒1^处的O-H伸缩振动、2 905 cm^‒1^处的C-H伸缩振动、1 114 cm^‒1^处的Si-O-Si吸收峰。这些特征峰的存在，共同证实POSS-epoxy结构已通过化学交联成功接枝于生物炭上，成功制备出BC@PEI-POSS杂化聚合物材料。

**图4:**
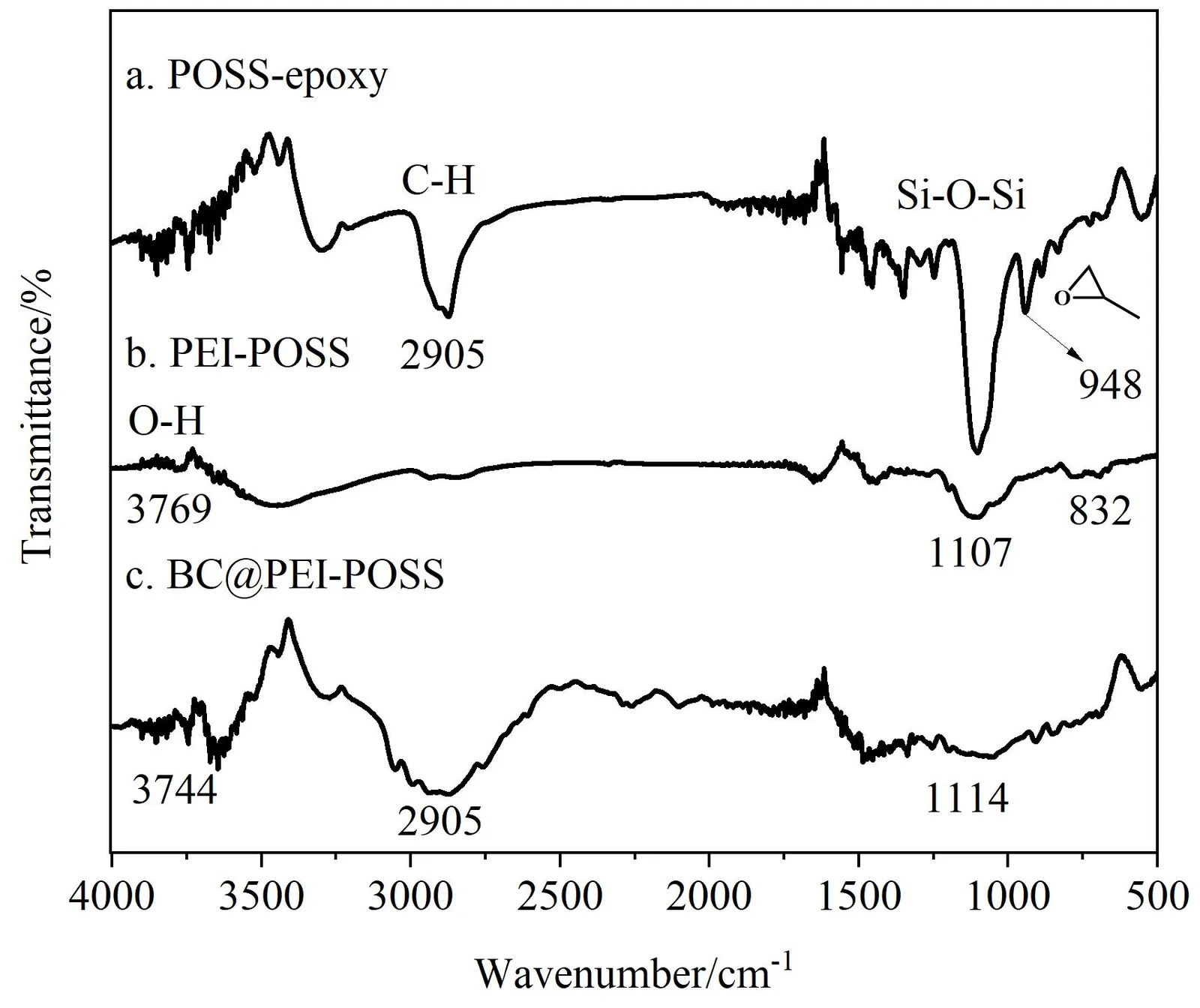
不同单体和聚合物的FTIR光谱图

#### 2.1.3 水接触角测试

采用悬滴法对水在BC@PEI-POSS功能整体材料表面的接触角进行了原位表征，结果如图5所示。由于PEI具有超亲水性，成功改性了原本疏水的POSS骨架，使得所制备的BC@PEI-POSS生物质衍生功能材料表现出良好的亲水特性，其接触角低至23.5°。

**图5:**
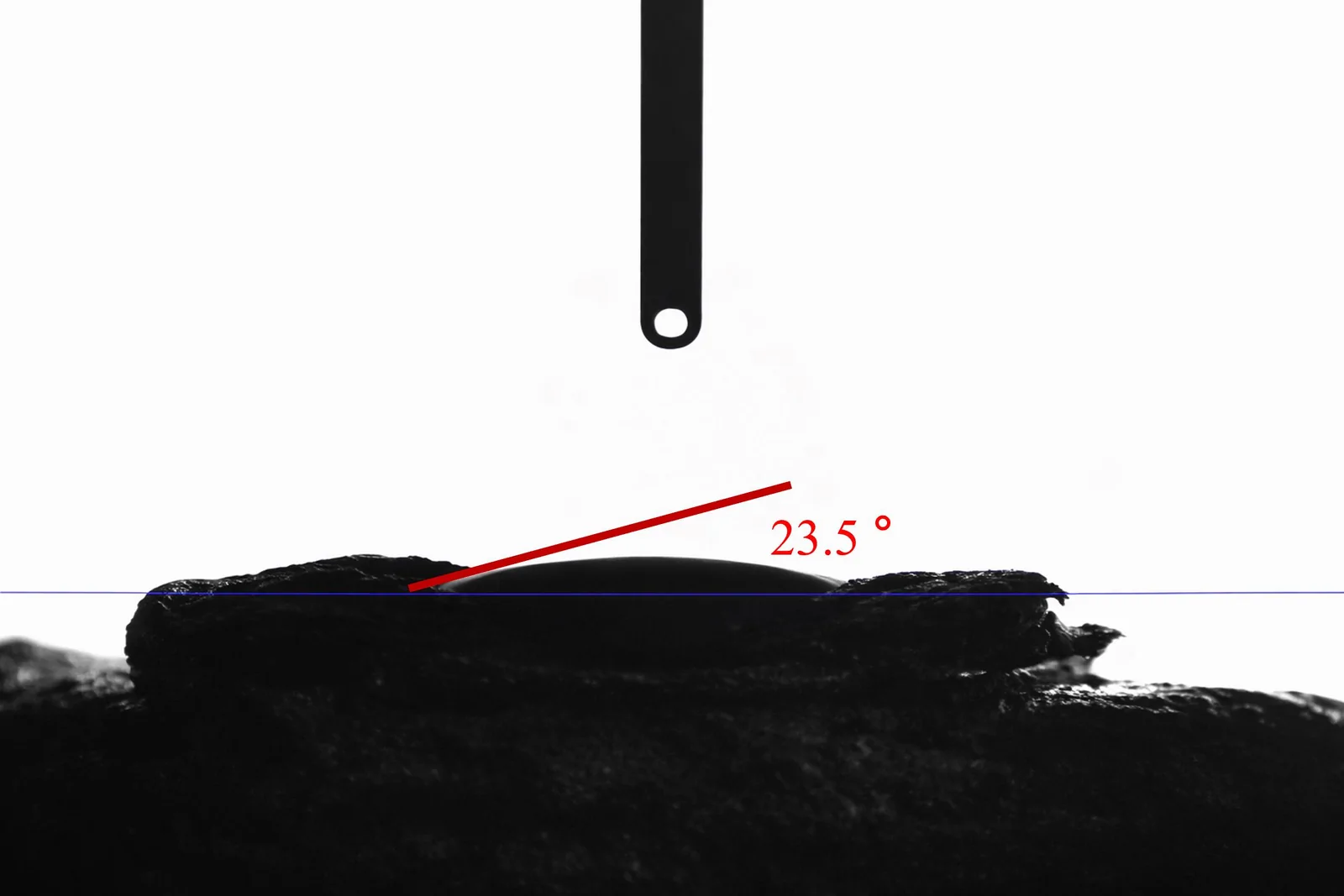
BC@PEI-POSS材料的水接触角分析

### 2.2 生物质衍生复合材料的潜在萃取机理

本实验所选目标物万古霉素与替考拉宁为糖肽类抗生素，其结构共有的特征官能团，如糖基、肽键、羧基及芳香环，为BC@PEI-POSS复合材料实现多重协同吸附提供了基础。具体地，PEI链上丰富的氨基可与抗生素的糖基及肽键形成氢键，在富集条件下，PEI中部分质子化的氨基也可与分析物带负电的羧基产生静电吸引作用。BC基底与POSS骨架共同提供了疏水界面，可通过*π-π*堆积作用有效捕获抗生素分子中的芳香环。此外，材料所具有的多级孔道结构有效促进了传质效率，从而利于吸附性能的提升。

### 2.3 影响PT-SPE萃取效率的关键参数优化

洗脱条件（例如，酸的种类与浓度、离心转速及洗脱体积）显著影响目标物抗生素的PT-SPE效率。如图6a所示，洗脱液中酸的种类是决定洗脱性能的关键因素。FA因酸性适中（pH≈2~3），能有效破坏分析物与吸附剂间相互作用，表现出最优回收率；HAc效果次之，这可能与其相对较弱的酸性（p*K*_a_≈4.76）有关，在相同浓度下，其质子化能力不足，难以充分解吸目标物；而TFA和PA则因强离子对效应，易出现色谱峰拖尾；此外，二者较高的黏度也降低了传质效率，这些因素共同导致了回收率的下降。因此，选择FA作为洗脱液中的酸性添加剂。

**图6:**
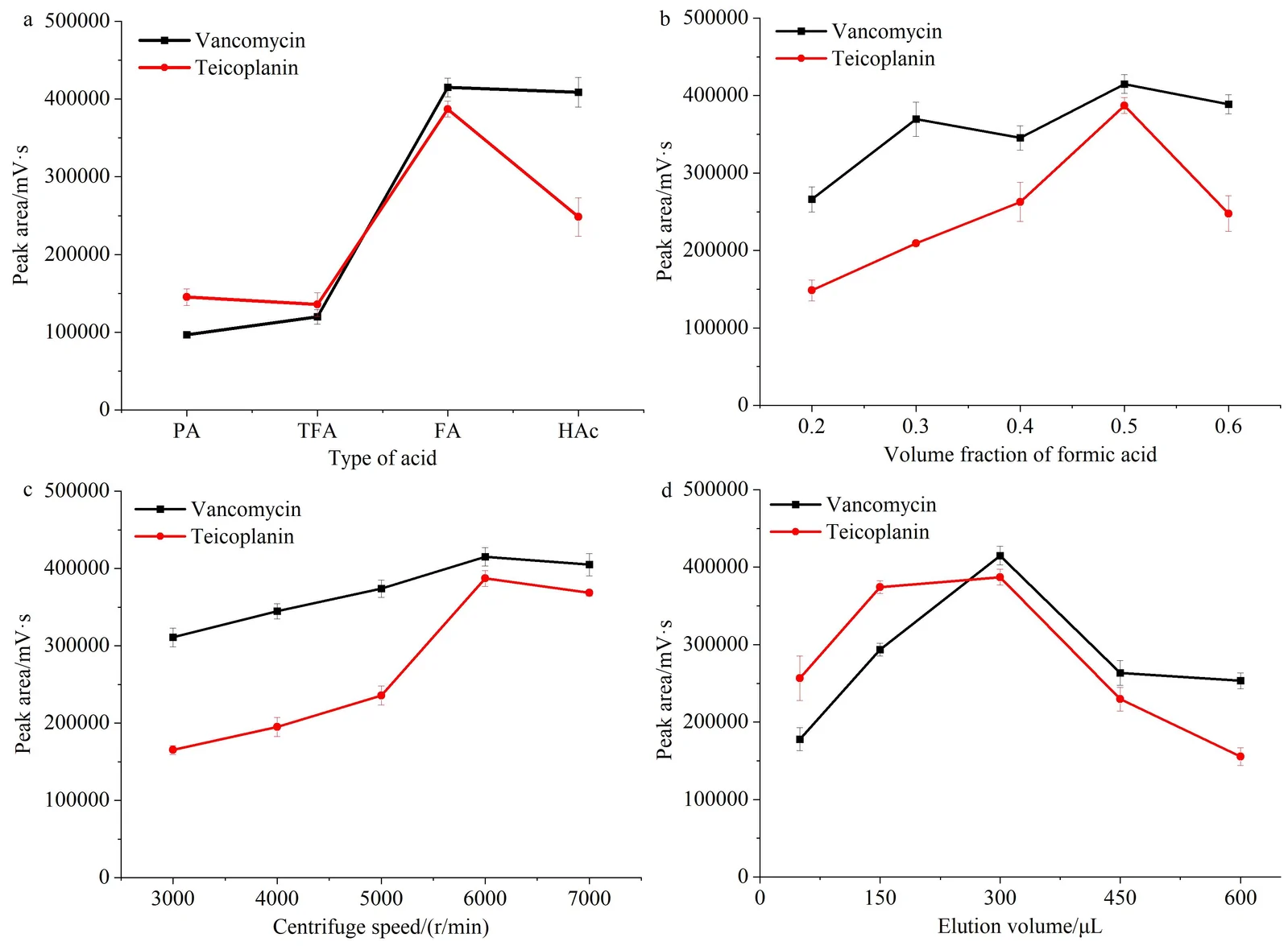
不同PT-SPE条件对目标分析物（25 mg/L）富集效率的影响（*n*=3）

FA在洗脱液中的含量也会对目标物的萃取效率产生较大影响。从图6b可以看出，当FA体积分数从0增至0.5%时，目标物的色谱响应随之升高；超过0.5%后，响应不再增加甚至略有下降，这可能源于过度质子化导致分析物与吸附剂间静电吸引减弱，故最终选择0.5%作为FA体积分数的最优值。

洗脱离心转速对解吸效率有重要影响。结果如图6c所示，目标物萃取后的响应值随着转速升高至6 000 r/min逐渐增加并达到最高值，进一步增至7 000 r/min时因其与吸附剂接触时间缩短从而导致效率下降。此外，洗脱液体积也会对目标分析物的萃取效率产生影响。如图6d所示，采用300 µL洗脱体积时可获得最高的色谱响应；体积过小（如50 µL）可能导致洗脱不完全，过大（如600 µL）则会造成样品稀释，均使响应降低。因此，最终选择以6 000 r/min离心转速进行洗脱，洗脱体积为300 µL。

### 2.4 方法学考察

将所建立的PT-SPE-HPLC-UV方法用于糖肽类抗生素万古霉素和替考拉宁的富集分析，并研究了该方法的分析性能参数，包括线性范围、检出限（LOD）、定量限（LOQ）和精密度（RSD）。结果表明，两种分析物在各自范围内（万古霉素：6.0~600 mg/L；替考拉宁：5.0~500 mg/L）均呈现良好的线性关系（*R*²>0.997 1）。万古霉素和替考拉宁的LOD（*S/N*=3）分别为1.97和1.68 mg/L，二者的LOQ（*S/N*=10）分别为5.64和4.79 mg/L。日内精密度通过连续进样3次进行测定，所得峰面积的RSD值分别为8.4%和9.5%，表明该方法具有令人满意的灵敏度与精密度。

### 2.5 实际样品分析

为了评估所建立的PT-SPE-HPLC-UV方法对于实际环境样品的适用性，选取汉江水样进行分析。首先，对实际水样直接进样分析，结果如图7（曲线Ⅰ）所示，万古霉素与替考拉宁的色谱峰响应极低，几乎无法检测。进一步比较不同吸附材料的性能，BC基PT-SPE材料（曲线Ⅱ）虽具备一定吸附能力，但难以同时对两种目标物实现高效吸附。这可能是由于其孔径分布较宽、表面化学性质不均一，导致对不同结构化合物的选择性吸附能力有限。相比之下，生物质衍生聚合物材料BC@PEI-POSS（曲线Ⅲ）表现出更优异且广泛的吸附性能，归因于其可调控的孔径结构以及POSS基团所赋予材料的刚性骨架和良好的机械稳定性，从而能够有效捕获并保留多种目标物。

**图7:**
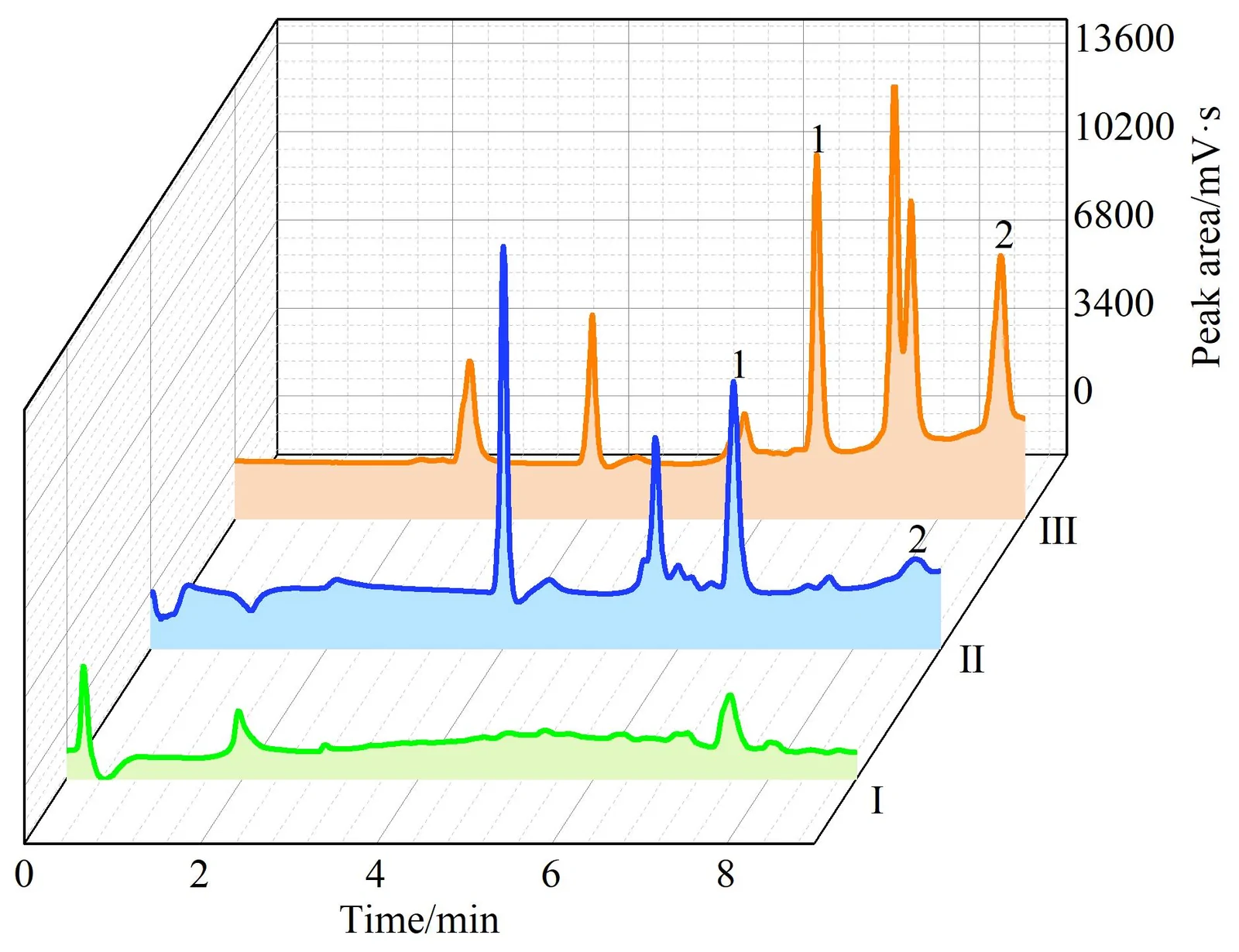
不同处理方式下加标水样中万古霉素与替考拉宁（20 mg/L）的HPLC-UV色谱图

如表1所示，采用所建立的PT-SPE-HPLC-UV方法开展加标回收试验，在水样中定量加入目标抗生素标准品储备液，配制成低、中、高3个水平（如50、150、300 mg/L）的加标样品，按照所述PT-SPE方法进行富集与洗脱，并通过计算回收率系统评价方法的准确性。结果表明，在不同加标水平下，目标物的平均回收率为93.6%~98.3%，所得峰面积的RSD值均低于9.2%，表明该方法在复杂实际样品分析中具有良好的准确性和可重复性。

**表1:** 实际水体样品中目标分析物在3个加标水平下的回收率（*n*=3）

Analyte	Added/ （mg/L）	Found/ （mg/L）	Recovery/%	RSD/%
Vancomycin	50	47.2	94.4	8.2
	150	147.5	98.3	6.5
	300	286.5	95.5	6.3
Teicoplanin	50	46.8	93.6	9.2
	150	146.9	97.9	5.4
	300	289.6	96.5	6.8

## 3 实验的组织实施及教学反思

### 3.1 实验的组织实施

本实验共计16学时，分为两次课程完成，每次8学时。第一次课主要内容为生物质衍生吸附剂的制备与初步表征；第二次课包括材料结构详细表征（如扫描电镜、红外光谱、接触角测试）、吸附性能实验、实际水样中污染物的固相萃取流程及液相色谱分析，最后进行实验总结与数据分析讨论。

在材料合成阶段，为了培养学生的科研设计与创新能力，我们赋予了学生一定的自主设计权。例如，学生可以自主选择生物质原料的种类（如咖啡渣、秸秆、甘蔗渣等）或表面活性剂PEG的分子质量作为变量。在表征与测试环节则安排2人一组协作进行，小组成员需共同商定并统一其组内的实验变量，以提高设备利用效率并培养团队协作能力。

### 3.2 实验问题反馈及注意事项

生物质衍生复合材料的合成过程耗时较长，且反应条件对材料性能影响显著。建议学生在实验前充分查阅相关文献，理解关键制备参数；在反应间隙可引导学生讨论绿色材料设计理念及环境分析中的应用前景，同时遵守实验安全规范。材料表征涉及多种大型仪器，为提高效率并深化理解，教师需在操作前系统讲解仪器原理、操作要点与数据分析方法。学生需提前预习仪器设备操作指南，实验中轮流操作、及时讨论和反馈现象，共同分析结果。固相萃取及色谱分析步骤较多，需合理分配时间与任务。建议小组内明确分工，教师重点讲解污染物萃取中的质量控制要点，避免操作失误影响实验结果。

此外，为使学生更全面理解材料设计的灵活性与应用潜力，本次实验在设计中特别扩展了碳基复合材料的构建思路。在原有生物炭的基础上，引导学生思考将生物炭与磁性纳米颗粒（如Fe₃O₄）、金属有机框架（MOFs）或黏土矿物等材料进行复合，以增强其在吸附、催化或分离等方面的性能。通过对比单一生物炭与复合材料的性能差异，学生能够更直观地认识到材料复合在优化功能、改善稳定性、提升污染物去除效率方面的作用，从而真正理解“为特定环境问题设计材料”的实验目的。

实验结束后通过问卷调查和小组讨论收集学生反馈。多数学生认为该实验将学科前沿与实用技术相结合，增强了科研兴趣和创新意识。通过完整参与材料设计、合成、表征、分析全流程，学生不仅掌握了多种仪器操作方法，还加深了对环境分析领域实际问题的理解。有学生表示通过自主选择原料或合成参数并观察其对吸附性能的显著影响，体会到科研的挑战与成就感。部分学生建议可进一步增加样品前处理技术的比较研究，以拓宽方法学视野。本实验在实施中也发现，部分学生在多环节衔接和时间管理上存在困难，今后将优化指导流程，引入阶段性总结与提示，帮助提升实验效率。总体来看，该开放性实验有效促进了学生综合科研素养的提升，为后续课程学习及科研实践奠定了基础。

## 4 结语

本研究构建了将生物质衍生复合功能材料应用于环境污染物分析的开放性实验，突破了仪器分析实验教学的传统模式。传统实验内容固定、流程预设，学生通常按指导书“照方抓药”，侧重于对理论知识的单一验证和仪器的基本操作，缺乏对科学问题探索的完整训练。该设计以科研前沿问题为导向，引导学生自主完成从材料设计、合成、表征到样品分析的全流程，着重培养其创新思维、分析和解决问题的综合能力及绿色化学意识。这一实践是对传统实验教学的重要革新，有效提升了学生的科研素养与实践能力。为持续深化教学效果，未来可进一步引入多元化新材料与复杂样品体系，推动实验教学与科研前沿更紧密融合，为培养高素质复合型创新人才构建行之有效的实践路径。
